# Effect of radiotherapy on freedom from seizures in dogs with brain tumors

**DOI:** 10.1111/jvim.15695

**Published:** 2020-02-07

**Authors:** Susana R. Monforte Monteiro, John H. Rossmeisl, Jason Russell, Mark A. Holmes, Annette Wessmann, Jo Morris, Jane M. Dobson, An E. Vanhaesebrouck

**Affiliations:** ^1^ The Department of Veterinary Medicine University of Cambridge Cambridge UK; ^2^ The Department of Veterinary Sciences Virginia Maryland College of Veterinary Medicine Blacksburg Virginia; ^3^ Department of Pharmacology Vanderbilt University Nashville Tennessee; ^4^ Pride Veterinary Centre Riverside Road Derby UK; ^5^ The School of Veterinary Medicine University of Glasgow Glasgow UK

**Keywords:** epilepsy, neoplasia, outcome, radiation

## Abstract

**Background:**

Seizures are a common presenting sign in dogs with brain tumors.

**Hypothesis/Objectives:**

To investigate the effect of radiotherapy on freedom from brain tumor‐associated seizures and survival time in dogs.

**Animals:**

Thirty‐two client‐owned dogs with brain tumor‐associated seizures; 18 received medical treatment and radiotherapy, 14 received medical treatment alone.

**Methods:**

Multicenter retrospective study. Baseline characteristics (seizure semiology, magnetic resonance imaging [MRI] characteristics, and treatment) and duration of seizure freedom were recorded for the 2 treatment groups. Duration of seizure freedom between groups was compared (log‐rank test) using Cox's proportional hazard analysis, with baseline characteristics entered as covariates.

**Results:**

The duration of seizure freedom and survival time were significantly longer in the radiotherapy group (*P* < .001), with a mean of 24 months (95% confidence interval [CI], 14.3‐33.8) versus 1.7 months in the control group (95% CI, 0.5‐2.9) and a mean of 34.6 months (95% CI: 25.2‐44.1) versus 6.2 months in the control group (95% CI, 2.6‐9.7) respectively. Baseline characteristics were not associated with duration of seizure freedom after the start of treatment. In the radiotherapy group, 5 dogs were euthanized during the study period because of causes other than seizures. In the control group, recurrence of seizures was observed before death in all dogs.

**Conclusions and Clinical Importance:**

A longer period of seizure freedom and longer survival time was observed in dogs with brain tumors after radiotherapy compared to medical treatment only. The pathophysiological mechanisms of epileptogenesis and the effect of radiation therapy on seizure control are unclear to date. Further prospective studies are needed.

**Abbreviations**
AED: antiepileptic drugs
MRI: magnetic resonance imaging

## 1 INTRODUCTION

Seizures are a common presenting clinical manifestation in dogs with brain tumors^1^ and are a major concern of the owners of affected dogs because they are perceived to greatly affect the animal's quality of life.

In veterinary medicine, control of seizure activity in patients with brain tumors is mainly based on the use of antiepileptic drugs (AED) and steroidal anti‐inflammatory drugs. Other treatment modalities reported in conjunction with medical treatment include surgical resection, chemotherapy or radiotherapy.^2^, ^3^

In human patients, there has been interest in investigating the role of surgical resection, chemotherapy and radiotherapy in seizure control.^4^, ^5^ Radiation therapy improves seizure control in humans, but no similar studies are available in dogs.^5^

Our aim was to investigate the effect of radiotherapy on freedom from seizures and survival time in dogs with brain tumors. We also investigated whether the clinical presentation of seizures, magnetic resonance imaging (MRI) characteristics of the lesions and drug dosage (AED and corticosteroids) would have confounded this outcome.

## 2 METHODS

### 2.1 Dogs

Medical records of dogs that presented between 2004 and 2011 with seizures in association with neoplastic intracranial masses were retrieved from 3 veterinary referral centers. The Queen's Veterinary School Hospital (University of Cambridge, United Kingdom [UK]) and the School of Veterinary Medicine (University of Glasgow, UK) provided the study population. Control data were acquired from a group of dogs treated medically for brain tumors in a previously published study from the Virginia Maryland College of Veterinary Medicine (United States of America).^2^

Study and control dogs were included if they fulfilled the following inclusion criteria: (a) clinical features of seizures and (b) MRI features of a brain tumor. All dogs underwent MRI of the brain and only dogs in which both a board‐certified radiologist and neurologist had described a brain tumor as the most likely differential diagnosis were included. The presumed diagnosis of brain tumor on MRI was based on lesion topography (ie, number of lesions, shape of the lesion, and presence of mass effect, dural tail [thickening of the dura adjacent to intracranial pathology on contrast enhanced T1‐W images] or contrast enhancement).^6^ Histopathological data were analyzed when available. Dogs were excluded if they underwent additional chemotherapy or brain surgery. The minimum follow‐up period for both groups was 12 months or until death or euthanasia before reaching the end of the 12‐month follow‐up period.

### 2.2 Radiotherapy and medical group

Dogs that received whole brain radiotherapy and medical treatment formed the radiotherapy group and dogs that received medical treatment alone formed the medical group. The radiotherapy course (using 6 MV X‐rays or 6‐15 MeV electrons) was variable, depending on institution, from 5 doses once weekly (1 × 5 Gy, followed by 4 × 8.25 Gy—total dose of 38 Gy) for 15 dogs, 12 fractions of 4 Gy each (total dose of 48 Gy) for 2 dogs or 15 fractions of 3 Gy each (total dose of 45 Gy) for 1 dog (Supplementary Data S1).

Medical treatment consisted of the use of AED (phenobarbital or levetiracetam) and corticosteroids (prednisolone; Table 1).

**Table 1:** Comparison of the baseline characteristics of the study population between both groups (radiotherapy and medical)

Variable	Radiotherapy group (n = 18)	Medical group (n = 14)	Statistics (*P* value) and statistical test used
Sex (male/female)	12 (67%)/6(33%)	7 (50%)/7(50%)	0.4727 (Fisher's exact test)
Age (years) at presentation	8.3 ± 3.25	8.9 ± 4	0.4832 (*t* test)
Breed (brachycephalic/other)	4 (29%)/10(71%)	8 (44%/10(56%)	0.4709 (Fisher's exact test)
Seizure type			
Generalized/Focal	18 (100%)/0(0%)	8 (57%)/6(43%)	0.0033 (Fisher's exact test)
Seizure presentation			
Cluster seizures	22% (4/18)	72% (10/14)	0.011 (Fisher's exact test)
Status epilepticus	0% (0/18)	14% (2/14)	0.1835 (Fisher's exact test)
None of the above	78% (14/18)	14% (2/14)	0.0010 (Fisher's exact test)
Seizure frequency prior to therapy			
1 seizure or cluster seizures before treatment	33% (6/18)	29% (4/14)	1 (Fisher's exact test)
> or = 1/month during last month before treatment	28% (5/18)	50% (7/14)	0.2769 (Fisher's exact test)
< or = 1/week during last month before treatment	28% (5/18)	21%) (3/14)	1 (Fisher's exact test)
> or = 1/week during last month before treatment	11% (2/18)	0% (0/14)	0.4919 (Fisher's exact test)
Tumor localization			
Intraaxial/extra‐axial	15 (83%)/3(17%)	6 (43%)/8(57%)	0.0265 (Fisher's exact test)
Lobe localization			
Frontal	28% (5/18)	29% (4/14)	1 (Fisher's exact test)
Temporal/Piriform	39% (7/18)	14% (2/14)	0.2349 (Fisher's exact test)
Parietal	0% (0/18)	14% (2/14)	0.1835 (Fisher's exact test)
Occipital	11% (2/18)	14% (2/14)	1 (Fisher's exact test)
Olfactory	17% (3/18)	22% (3/14)	1 (Fisher's exact test)
Other	5% (1/18)	7% (1/14)	1 (Fisher's exact test)
Presence of perilesional extension into another lobe	50% (9/18)	50% (7/14)	1 (Fisher's exact test)
Edema			0.054 (Mann–Whitney *U* test)
Mild	28% (5/18)	29% (4/14)	
Moderate	28% (5/18)	36% (5/14)	
Significant	44% (8/18)	14% (2/14)	
None	0% (0/18)	21% (3/14)	
Contrast enhancement			0.1679 (Mann–Whitney *U* test)
Mild	44% (8/18)	7% (1/14)	
Moderate	11% (2/18)	36% (5/14)	
Significant	28% (5/18)	43% (6/14)	
None	17% (3/18)	14% (2/14)	
Presence of cystic structures	67% (12/18)	43% (6/14)	0.2831 (Fisher's exact test)
Presence of brain herniation	11% (2/18)	36% (5/14)	0.1948 (Fisher's exact test)
Dilation of the ventricular system	11% (2/18)	36% (5/14)	0.1948 (Fisher's exact test)
Tumor type			
Glioma	78% (14/18) (based on MRI appearance)	36% (5/14) (confirmed)	0.0293 (Fisher's exact test)
Meningioma	17% (3/18) (based on MRI appearance)	50% (7/14) (confirmed)	0.0623 (Fisher's exact test)
Other	5% (1/18) (based on MRI appearance)	14% (2/14) (confirmed)	0.5681 (Fisher's exact test)
Mass‐effect	3.1 mm (median = 2.65, IQR = 3.68)	2.5 mm (median = 2, IQR = 4.25)	0.5360 (Mann‐Whitney *U* test)
Brain:lesion size ratio	0.31 (median = 0.32, IQR =0.118)	0.31 (median = 0.31, IQR =0.155)	0.8958 (Mann‐Whitney *U* test)
Medical treatment			
Phenobarbitone	94% (17/18) median max dose of 6.6 mg/kg/day	100% (14/14) median max dose of 6.45 mg/kg/day	1 (Fisher's exact test)
Other AED	6% (1/18)	0% (0/14%)	1 (Fisher's exact test)
Corticosteroids (prednisolone)	94% (17/18) mean max dose of 0.84 mg/kg/day	79% (11/14) mean max dose of 0.87 mg/kg/day	0.2951 (Fisher's exact test)

*Note: t* test, Mann‐Whitney *U* test, and Fisher's exact test were used depending on the variable's characteristics. Due to multiple comparisons the Bonferroni correction was applied, with statistical significance being established at *P* < .002.

### 2.3 Retrieved data

Pretreatment data included age, sex, breed, and time between diagnosis and start of radiotherapy. In addition, onset of seizure activity, seizure frequency (divided into: [1] 1 seizure episode, [2] ≥1 seizure episode per month and < 1 per week, [3] ≥1 per week and < 1 per day, [4] and > 1 per day), seizure type (focal [based on history, owner's description and video when available] or generalized); presence of cluster seizures (≥2 seizure episodes within 24 hours) or status epilepticus (continued seizure activity >5 minutes or multiple seizures for >30 minutes with impaired consciousness between seizures); presence of other neurological signs; and, dosage of AED, corticosteroids or both given before radiotherapy. A seizure episode was defined as a single seizure or a cluster of seizures.

Magnetic resonance imaging studies included a minimum of T1‐W (pre‐ and post‐contrast) and T2‐W sequences in transverse and at least either sagittal or dorsal planes. Fluid‐attenuated inversion recovery (FLAIR) and gradient echo (GRE) sequences also were included in most studies. The MRI characteristics of each suspected tumor were assessed according to the following criteria: (a) whether the neoplasia was intra‐axial or extra‐axial, (b) lesion localization (frontal, temporal/piriform, parietal, occipital, olfactory, or other), (c) presence or absence of perilesional extension into a different lobe, (d) presence of edema or contrast enhancement (both subjectively classified as none, mild, moderate, or severe), (e) presence or absence of cystic structures, and (f) presence or absence of brain herniation or dilatation of the ventricular system. The height of the lesion divided by the height of the brain at its highest point (measured perpendicular to the base plane of the skull) was calculated to determine a relative ratio of the tumor to brain size. Any mass effect was calculated as the largest deviation from midline in transverse sections. All measurements on MRI were made using a computer software program (Visbion) by one of 2 of the authors (SM and JHR) on T1‐W postcontrast images or T2‐W images, if no contrast uptake was observed. A tentative diagnosis of brain tumor type was made based on the MRI findings. Definitive diagnosis by histopathological analysis was recorded, if available.

After radiotherapy, the owners were contacted at the end of 2012 and, according a standard questionnaire, asked whether the animal's seizures were controlled (defined as <1 seizure per month) before treatment and to grade the animal's seizure frequency at 3, 6, and 12 months after treatment as part of clinical follow‐up. The owners were not made aware of the purpose of the study. When available, additional records about seizure activity of the patient before and after initiation of treatment were reviewed. If the animal had been euthanized, the owner was asked whether euthanasia was related to seizure activity. The time when the animal was euthanized was recorded. For each dog, seizure freedom was calculated, defined as the duration of time that the dog remained seizure‐free either since the start of radiotherapy for the radiotherapy group or since the start of the drug treatment for the medical group. Corticosteroid and AED dosages during the course of medical treatment and radiotherapy also were recorded (Table 1).

To identify errant variables (those nonrelated to treatment) that might predispose patients to achieve seizure freedom, data from both groups were compared, including patient signalment, clinical presentation (seizure characteristics), drug choice and dosage and MRI findings (Table 2). Baseline characteristics of the 2 groups also were compared (Table 1).

**Table 2:** Cox proportional hazard analysis on selected variables, evaluating association with seizure freedom

Variable	Statistics (*P* value)—Cox's proportional hazard analysis for association of parameter with seizure freedom
Group	.001
Seizure type	.14
Seizure presentation	.43
Seizure frequency prior to treatment	.73
Brain:seizure size ratio	.59
Edema	.49
Tumor localization	.80
Mass effect	.49

### 2.4 Statistical analysis

The radiotherapy and medical treatment groups were compared for seizure freedom and survival time, using a Kaplan‐Meier Survival analysis. The level of significance was set at .05. Patients were censored if lost for follow‐up after 12 months, or if they died of unrelated causes (the latter for assessment of the seizure freedom).

Student *t* test, Mann‐Whitney *U* test, and Fischer exact test were used to compare baseline characteristics of both groups. The Bonferroni correction was applied to correct for multiple comparisons, with statistical significance established at .002.

A Cox's proportional hazard analysis was used to evaluate whether the treatment effect on duration of seizure freedom could have been confounded by group covariates, such as signalment, clinical presentation, MRI characteristics, or AED dosage. The level of significance was set at .05.

## 3 RESULTS

### 3.1 Population characteristics

Thirty‐two dogs met the inclusion criteria: 18 dogs in the radiotherapy group, and 14 dogs in the medical group. No differences in baseline characteristics were observed between groups, except for seizure presentation, with single seizure episodes (*P* = .001) being overrepresented in the radiotherapy group (Table 1). However, Cox hazard proportional analysis (see later) did not identify any variables cofounding the outcome (seizure freedom).

### 3.2 Comparison of seizure freedom and survival time between radiotherapy and medical groups

**Figure 1:**
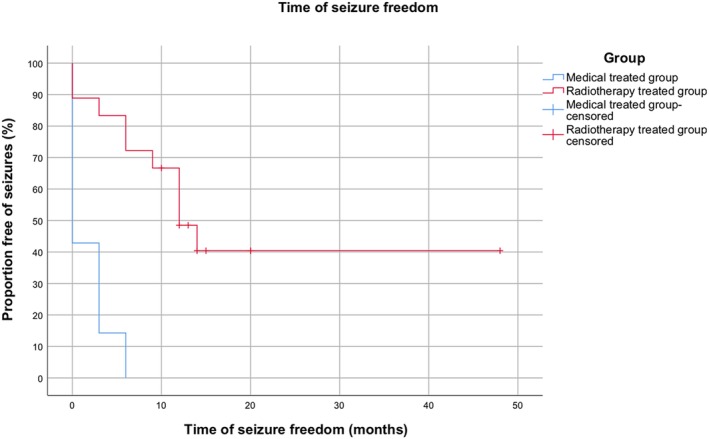
Kaplan–Meier event‐free survival curves, comparing the cumulative percentages of dogs in the 2 groups who were free of seizures. Dogs of the radiotherapy group (red) remained free of seizures dogs for longer as compared to dogs of the medical group (blue), following the start of therapy. Note that time 0 is defined as the start of radiotherapy for the radiotherapy group or the start of the drug therapy for the medical group

**Figure 2:**
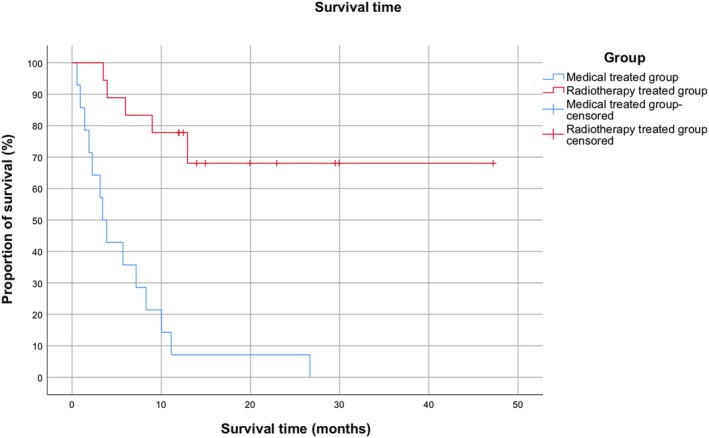
Kaplan–Meier survival curves, comparing the cumulative percentages of dogs in the 2 groups. Dogs of the radiotherapy group (red) had a longer survival time as compared to dogs of the medical group (blue), following the start of therapy. Note that time 0 is defined as the start of radiotherapy for the radiotherapy group or the start of the drug therapy for the medical group

A statistically significant difference in freedom from seizures (*P* < .001) was found between the 2 groups, with the radiotherapy group showing markedly longer periods of seizure freedom. The radiotherapy group had a mean seizure‐free time of 24 months (95% CI, 14.3‐33.8) versus 1.7 months in the control group (95% CI, 0.5‐2.9; Figure 1). Survival time also was significantly longer in the radiotherapy group (*P* < .001), with a mean of 34.6 months (95% CI, 25.2‐44.1) versus 6.2 months (95% CI, 2.6‐9.7) in the control group (Figure 2).

Nearly half (44.4%; 8/18) of the dogs included in the radiotherapy group still were seizure‐free by the end of the study, versus none (0/14) of the medical group (Table 3).

**Table 3:** Comparison of seizure freedom at different times between the radiotherapy and medical groups

Seizure freedom	Radiotherapy group (n = 18)	Medical group (n = 14)
Seizure free time < 3 mo	11% (n = 2)	8 (57%)
Seizure free time 3–6 mo	1 (6%)	4 (29%)
Seizure free time 6–9 mo	2 (11%)	2 (14%)
Seizure free time 9‐12 mo	2 (11%)	0 (0%)
Seizure free time > 12 mo	11 (61%)	0 (0%)
Seizure free by end of study	8 (44.4%)	0 (0%)

In the radiotherapy group 5/18 dogs (27%) were euthanized because of causes other than seizures. One dog was euthanized because of anemia, another dog because of lymphoma (unrelated to the central nervous system) and 3 dogs because of development of other neurological signs. The surviving dogs with recurrence of seizures had less severe seizure forms (changing from generalized to focal) or only 1 or 2 episodes after the course of radiotherapy (Figure 2).

In the medical group, all the dogs had died or been euthanized by the end of the study, 71% (10/14) because of seizure activity and 29% (4/14) because of the development of other neurological signs.

### 3.3 Confounding variables

A Cox proportional analysis was used to explore whether signalment, clinical presentation, MRI characteristics or drug dosage could have confounded the time of seizure freedom after radiotherapy. None of the explored characteristics was found to be associated in a statistically significant manner with time of seizure freedom after intervention (Table 2). Only group (radiotherapy versus medical) was significantly different (*P* = .001).

## 4 DISCUSSION

To our knowledge, this study is the first to investigate the potential of radiotherapy to prolong seizure freedom in dogs with brain tumors, and suggests that radiotherapy decreases seizure frequency in dogs. Similar findings have been observed in humans patients with brain tumors treated with radiotherapy.^5^ This information is crucial for the owner's decision making when choosing whether to pursue further treatment or not, because often recurrence of seizures is a common reason for euthanasia^7^ in these patients. Interestingly, in our study 70% of the dogs in the medical group were euthanized because of seizure activity rather than other neurological impairment, which could indicate the importance of seizure freedom on the perceived quality of life of the dog for the owners. Similar findings have been described previously.^7^

The significant difference between seizure‐free duration in the radiotherapy group as compared to the control group allows radiation therapy to be presented to owners as a potential aid in seizure control in dogs with brain tumors. However, because ours was a retrospective study, prospective studies are warranted to confirm this observation.

The reason for the increased duration of seizure freedom after radiotherapy observed in our study remains unclear. In a study of humans with gliomas^5^ a significant difference in seizure freedom was achieved at 12 months in 32%‐38% of patients. Only the duration of seizures before radiotherapy influenced outcome: Patients with a seizure history of >1 year before radiotherapy had a worse outcome.^5^ No significant correlation was found between patients with seizure reduction and reduction of tumor size after radiotherapy.^5^ In our study, tumor size after radiotherapy was unknown, because the owners declined repeated MRI. Based on extrapolation from studies in humans, we speculate that the role of radiotherapy in seizure control is not related directly to tumor size, and it is postulated that another mechanism likely plays a role, particularly in those patients with no tumor size reduction.^5^

Epileptogenesis in patients with brain tumors still is not well understood. Tumors in the frontal, temporal, and parietal lobes and in the olfactory bulb have been associated with an increased incidence of seizures.^1^ The epileptogenic zone often is not restricted to the tumor area. Perilesional edema also is implicated in the mechanism of epileptogenesis.,^1^, ^8^ In fact, in approximately one‐third of the human patients, the epileptogenic zone is not within the tumor itself, but in the surrounding brain tissue.^9^ Seizures in humans generally are associated with low‐grade tumors, suggesting that slow rate growth might allow time for development of functional (rather than structural) changes to occur in the tumor cells and surrounding tissue.^10^

The use of radiation therapy, although aiming to destroy the neoplastic cells, also damages surrounding brain tissue. Because the peritumoral area is the most likely site for the seizure focus, the effect of radiotherapy on the myelin and thus electrical conduction, and also on altered neurons, conceivably could eliminate the functional deficits.^11^ This effect could lead to eliminating the source of the hyperexcitability, and therefore the seizures, without a marked decrease in lesion size. Further studies would be necessary to validate this hypothesis.

Our study had a number of limitations. Only the control group had histologically confirmed tumors. None of the dogs in the radiotherapy group that died had necropsy performed, thus the exact histological origin of the presumed tumor on MRI remains unknown. It is known that MRI has a sensitivity of approximately 90% and specificity of approximately 87% for correct identification of brain neoplasia.^10^ It also has been shown, however, that cerebrovascular incidents can be mistaken for brain tumors.^6^ Using diffusion‐weighted sequences would be an appropriate method to differentiate between tumor and cerebrovascular incident. In veterinary practice, most animals with brain tumors are irradiated based on MRI findings, and most owners are not willing to have a brain biopsy performed on their animal. The MRI characteristics of the tumors were not significantly different between groups. The time and choice of euthanasia also might contribute bias, because owners electing radiotherapy might be more motivated and euthanize their dogs later when compared to owners who have chosen medical treatment alone. The decision for euthanasia in dogs with symptomatic epilepsy is largely dependent on the owner's personal view on the animal's quality of life.^7^ As a retrospective study, some of the radiation therapy protocols differ from most current ones.^12^ Our study included a control group from a different time period and location (USA) than the radiotherapy group, which increases the risk of confounders. The demographics of the 2 populations were similar with the exception of seizure presentation, with dogs in the radiotherapy group being presented mainly with single seizures. None of the variables recorded, however, had a significant association with freedom from seizures, with the exception of treatment modality. Other unrecorded characteristics related to the geographic differences between populations, such as diet and environment, could have an influence. The time between presenting seizure activity and the collection of information from the owners may have led to some recall bias. It would have been useful to evaluate the efficacy of radiation therapy in decreasing seizure frequency by comparing seizure frequency before and after treatment between the 2 groups. Doing so was not possible in our study because intervention (both medical or radiotherapy) was started soon after the onset of seizures and diagnosis. A prospective study could further explore this question.

In conclusion, in our study a longer period of seizure freedom was observed in dogs with brain tumors after radiotherapy compared with those that received medical treatment alone. These findings encourage performance of a prospective multicenter study to confirm our results. Such information might help owners in their decision whether to pursue radiotherapy as a treatment option for animals with suspected brain tumors. Further prospective case‐controlled, blinded, randomized studies are needed in humans and dogs to investigate underlying pathophysiological mechanisms, because tumor size and shrinkage and severity of edema do not seem to be the only reasons for the decrease in seizure frequency.

## CONFLICT OF INTEREST DECLARATION

Authors declare no conflict of interest.

## OFF‐LABEL ANTIMICROBIAL DECLARATION

Authors declare no off‐label use of antimicrobials.

## INSTITUTIONAL ANIMAL CARE AND USE COMMITTEE (IACUC) OR OTHER APPROVAL DECLARATION

Authors declare no IACUC or other approval was needed.

## HUMAN ETHICS APPROVAL DECLARATION

Authors declare human ethics approval was not needed for this study.

## Supporting information


**File S1**: Supporting Information
