# Supplementary material for: Effect of radiotherapy on freedom from seizures in dogs with brain tumors
Source: J Vet Intern Med. 2020 Feb 7;34(2):821–7. doi: 10.1111/jvim.15695 (PMC7096644; doi:10.1111/jvim.15695)
Supplement: Supplementary file 1 — File S1: Supporting Information [file JVIM-34-821-s001.pdf]

Radiotherapy protocols description

All dogs were treated with external beam megavoltage radiation delivered with a 6-MV (megavoltage) linear accelerator (VARIAN, Clinac DMX-Varian Medical Systems UK LTD in Cambridge and Siemens Oncor Impression Lin Acc in Glasgow). The linear accelerator was not equipped with a multileaf collimator or portal imaging in Cambridge. In Glasgow the linear accelerator was equipped with a 80 leaf MLC with 1cm leaves and portal imaging. Treatment fields varied in size, from 3x3cm and 4x4cm in Cambridge to up to 3.8x5cm in Glasgow. 6-MV photons were prescribed to the isocenter (the centre of the tumor). Each dog underwent anaesthesia to enable positioning and restraint during radiotherapy. Photon beams were delivered in three portals (one dorso-ventral and two opposing laterals) in Cambridge, and a conjunction of up to four portals in Glasgow (dorso-vental, right and left lateral, dorsal right oblique and left dorsal oblique). The beams were calculated using a computer-aided planning system. The planning system (Addenbrookes Hospital Radiation Planning System-ARPS in Cambridge and Prowess Panther v4.71 in Glasgow) was used to produce a 2 dimensional isodose plot of predicted radiation distribution on the selected transverse MR image or CT image of the dog's head. Patients were positioned in sternal recumbency and anatomical landmarks in the head (eg.: zygoma, ear canal) and skin to surface distances, as calculated by ARPS, were used to verify the dose delivery to the isocenter for each treatment. Gross tumor volume (GTV) was defined by contrast-enhancing areas on MR images (T2 study) or the outline of the tumour on CT images. Planning target volume (PTV) was defined by a margin of 5 mm around the GTV to account for patient's movement and positioning errors.

One of the patients from Glasgow had Mannitol prior to anesthesia at the CT planning, but not routinely during radiotherapy.

Owners' Questionnaire

1. How well controlled was <dogs name> seizures with anti-epileptic drugs prior to radiotherapy?
    a. Controlled
    b. Uncontrolled
2. Following radiotherapy how would you describe <dogs name> seizure frequency[1]?
    a. At 3 months
        i. Seizure-free
        ii. Rare-seizures
        iii. Meaningful improvement
        iv. No improvement
    b. At 6 months
        i. Seizure-free
        ii. Rare-seizures
        iii. Meaningful improvement
        iv. No improvement
    c. At 12 months
        i. Seizure-free
        ii. Rare-seizures
        iii. Meaningful improvement
        iv. No improvement
3. Is <dogs name> still alive? If not was he euthanised due to seizure activity?
